# A Biofunctional Perspective on Learning Environments

**DOI:** 10.3389/fpsyg.2015.01973

**Published:** 2015-12-23

**Authors:** Ryan Alverson

**Affiliations:** Department of Teacher Education, College of Education and Human Services, Northern Kentucky University, Highland HeightsKY, USA

**Keywords:** biofunctional understanding, learning environments, learning sciences, principles of learning, embodied cognition

## Abstract

This paper proposes that the principles of learning, as highlighted by Bransford and colleagues, require biological grounding. This process essentially amounts to the facilitation of, and development of, an enriched capacity for ongoing biofunctional activity (OBA)—physical biology in action. The learning sciences’ principles of active learning, i.e., accessing prior understanding, organizing knowledge through schemas, and self-monitoring are psychological-understanding vehicles that work only in the ground of biofunctional (deep) understanding. Biofunctional understanding is a form of understanding that rises out of OBA. In this sense, biofunctional understanding juxtaposes and inherently complements psychological understanding. Without biofunctional understanding, psychological understanding becomes synonymous with superficial understanding. By contrast, in the illuminating context of biofunctional understanding, psychological understanding enables accomplishments of the kind enjoyed by experts in the rich and deep, metaphorically speaking, experiences of their respective fields of specialization. In this perspective article, I show how and why the psychological principles of active learning are ready for grounding in biofunctional understanding.

However, the external environment is not the only immediate source of knowledge, and the process of acquiring 2nd/3rd-person knowledge is not the only kind of knowledge acquisition. A second type of knowledge acquisition is 1st-person knowledge acquisition. The immediate source of 1st-person knowledge is biology, in general, and biofunctional understanding, specifically. Therefore, 1st-person knowledge acquisition demands a qualitatively different type of relationship with the external learning environment, whether this biofunctional learning environment is built into the traditional classroom or the external environment as a whole. At this point, it is necessary to distinguish 1st person education from 2nd/3rd person education (Iran-Nejad, 2013) to delineate traditional educational approaches from biofunctional approaches.

## Psychological Learning Principles that Stand Out Among Others

Learning from an embodied (1st person) view means that humans are inherently designed to learn through a dynamic, multiple-source process, that involves pulling from affective, cognitive, and behavioral resources (i.e., OBA), in which they engage in a natural process of reflecting on their own intuitions (Iran-Nejad, 2000; Iran-Nejad and Gregg, 2011). A 1st person approach to education involves coordinating the educational infrastructure so that these natural learning resources are utilized at their maximum potential (Iran-Nejad, 2013). Counter to a 1st person approach, learning from a non-embodied (2nd/3rd person) view posits that students are given information from other people to digest. This process is known as internalization (Vygotsky, 1978). Teachers and other educators using a non-embodied (2nd/3rd person) approach, arrange the educational environment in ways that make internalizing knowledge, i.e., absorption of knowledge into the brain through conduit, “easier” (Iran-Nejad, 2013). In contrast, the 1st person, biofunctional approach originates first and foremost in biology, and then and only then from the environment and the others in it.

There are diverse psychological understanding theories in the interdisciplinary field of cognitive science. Information processing theory (Atkinson and Shiffrin, 1968) and Piaget’s (1952) theory of cognitive development are two better-known examples of these theories. In this article we have turned to the learning-sciences principles of Bransford and his colleagues (Bransford et al., 2000) because we find them directly in tune with the tenet of the theory of biofunctional understanding—they are more biofunctional-understanding ready than any other psychological perspective we know. In proposing that the core principles of the learning sciences are based on biofunctionally embodied understanding, we are suggesting that the builders of learning environments, who support these principles of active learning, should enlist and emphasize the biofunctional and dynamic nature of human learning and understanding, in order to promote higher levels of achievement in children and other learners. In addressing the ways in which educators may begin to address the principles of learning through a biofunctional lens, this paper hopes to stray from such traditional conduit metaphors of learning processes (Reddy, 1979), e.g., storing external knowledge and organizing external knowledge in long-term memory. Rather, it intends to focus on terms and phrases more readily agreeable to the embodied nature of learning.

## Assumptions About Learning Environments

This section attempts to set apart the learning sciences’ research investigated by Bransford and his colleagues from mainstream conduit-metaphor-laden information processing theory. The very idea of information processing theory is a conduit-metaphor perspective, or more accurately, a nest for conduit metaphors. Conduit metaphors describe learning as a process whereby the learner receives knowledge through an overall medium from the more knowledgeable expert. The specific metaphors employed by this overall medium are aligned with 2nd/3rd person approaches to education, which is probably why neither the information processing perspective, nor 2nd/3rd-person education have been able to climb into the realm of the higher cognitive functioning that enables deep understanding (Bransford et al., 1977; Iran-Nejad, 1990, 2013). The following sections discuss the principles of learning, first through mainstream theory, and secondly through biofunctional theory.

## Understanding through Principles Of Learning: Not a Unified Perspective

Research in the learning sciences holds that effective learning environments should be structured around three main principles in which people learn. These principles of learning are engaging prior understanding, organizing lived experiences, and self-monitoring. It is understood that when these environments are well-aligned with these three key principles, a unified perspective is born as essential. It follows that effective learning environments need to be structured around the learner, the learner’s lived experiences, and the learner’s self-regulation (Bransford et al., 2000). And this is exactly what 1st-person education is about. Each of these principles of learning environment design are intended to align with the principles of learning, so that students may learn effectively when placed in such environments. Effective learning, in this sense, is “deep” learning or understanding, rather than surface learning or understanding. When put into practice, commonly held assumptions regarding these principles rarely make it past simple metaphors, which are easily digested by educators and students. Examining each of the principles of learning, in turn, may highlight this effect.

“Engaging prior understanding”, as a phrase, has inherent tones of linear processing. Some view this type of linear thinking as segmental in nature (Iran-Nejad and Gregg, 2011). It is as if the learner must somehow connect what they are trying to learn with what they already know, and by doing so, he or she builds new knowledge on top of what already exists. Essentially, the old knowledge creates a foundation on which the new knowledge builds. With a firm enough foundation, the new knowledge is able to be effectively stored on top of the previous knowledge in long-term memory, e.g., bin theory (Wyer and Srull, 1981). This process needs a facilitator. Typically, it is the teacher who facilitates this process making sure students are thinking about what they already know regarding a particular concept, and assessing this prior knowledge. This type of functioning is well-aligned with a 2nd/3rd person learning perspective. The learner may also facilitate this process, provided they have learned the how and the value of doing so.

“Organizing knowledge” also has tones of linear processing, on one hand, and is often confused with organizing lived experiences on the other. It is assumed that the learner’s knowledge must be well organized in order for deep understanding to take place, thereby “transforming factual information into usable knowledge” (Bransford et al., 2000, p. 16). The better this knowledge is organized, the more expert-like the learner becomes. This already implies higher order thinking, involvement of knowing-with metacognition, fluid access to anchored (1st-person) conceptual understanding, and holistic engagement of context. Contrariwise, it also suggests little more than segmental information processing for accumulation in long-term memory (Iran-Nejad and Gregg, 2011), in which knowledge exists in the form of static building blocks of cognition. Left to their own devices or those of 2nd/3rd-person educators, novice learners simply never develop enough blocks nor do they ever rise clear of inert knowledge and into the realm of higher-order thinking. By contrast, experts have a rich wealth of conceptual knowledge pertaining to a particular discipline, and have them arranged in such a way that they may easily access any concepts they choose. Experts know which blocks of their knowledge, so to speak, are relevant and which ones are not needed for any particular task in which they are engaged. At the same time, linear information-processing theory is aligned with the conduit metaphor (Reddy, 1979), in which new knowledge is stored in layers on top of old knowledge. Even once the learner has successfully internalized the knowledge, it is unclear how top-to-bottom layers are navigated (Iran-Nejad, 1990) or how the teacher serves as facilitator of this process, e.g., by resorting to awkward concept maps and other cognitive tools in a learner-relevant fashion.

“Self-monitoring”, a metacognitive process, is the third principle of learning. Experts possess this important skill, whereas novices do not. This phrase means to take charge of one’s learning. A seminal example of this process would be reciprocal teaching (Palinscar and Brown, 1984), in which readers begin to internalize the process of monitoring their reading comprehension through asking questions, summarizing, and making predictions of the text. Additionally, this process should be taught in different contexts, since practicing it in general will not lead to transfer. The facilitator of this process is usually the teacher at first, until the learner is able to do it on his or her own. “Self-monitoring” works to enable the learner to regulate the aforementioned processes of “organizing knowledge” and “engaging prior understanding.” Learning this important set of skills is essential for deep understanding of the subject matter, but so far a mystery in information processing conduits.

These three principles of learning may operate together to promote meaningful learning and deep understanding. A distinction exists, however, regarding the approaches educators take when utilizing these principles in a learning context. From the 2nd/3rd person, non-embodied approach, educators take on the role of helping the learner along the way, giving them concepts to internalize, until he or she understands the process and is able to function on his or her own. From the 1st person, embodied approach, the educator facilitates the process of using the principles in a way that draws on the natural capabilities of the learner. The latter approach, and its relation to the principles of learning, is discussed next.

## A Wholetheme Take on Biofunctional Learning Environments

Given the previously stated assumptions regarding the principles of learning, and the ways they function within a learning environment, a biofunctional explanation is proposed in order to better understand the global coherence context of educational practice. This attempt at reconciliation between the commonly held assumptions regarding learning and biofunctional understanding will highlight how biofunctional activity governs these processes.

A compelling framework for a biofunctional explanation of learning environments is the 1st-person approach to education (Iran-Nejad and Stewart, 2011; Iran-Nejad, 2013). It makes more sense from a biofunctional approach to explain the global coherence context, in which the multiple sources and systems integrate, from a wholetheme, 1st-person perspective. This perspective asserts that the biological foundation of the learner must be at the forefront of understanding and promoting the learning process. Where the common assumptions regarding the principles of learning incorporate executive control as a regulating force within the learner, dynamic internal self-regulation involves non-executive components of the body that allow “spontaneous mental functioning” to occur (Iran-Nejad, 1990), free from conscious thought. An early study by Bransford and Johnson (1972) highlighted how learning, sentence semantics in this case, does not happen in isolation, but rather in the context of the learner’s prior knowledge. It also illustrated the non-segmental nature of learning. This type of natural functioning is the type of spontaneous mental functioning suggested by Iran-Nejad (1990).

So, the question now becomes, how does the active learner begin to understand, using a system over which he has no deliberate immediate (or direct) control? Here is where a unique role for learning environments comes into place (Iran-Nejad, 2013). One explanation is that it can occur in a suitable environment for the engagement of “spontaneous mental functioning,” to be under (indirect) control of the deliberate learner and eventually his or her integrated biofunctional and psychological understanding. If this is the case, then the teacher’s role as a facilitator of the learning process must change. Before, the teacher was the giver of information. Now, the teacher becomes a positioner of the learner. That is, the teacher positions the learner in an environment suitable for dynamic self-regulation to occur.

## Creating Biofunctional Classrooms

Having discussed the principles of learning through both mainstream and biofunctional lenses, this paper now discusses the implications for educational practice based on the biofunctional approach. One of the key challenges associated with this approach to education is the design of environments “that are rich with revelation-learning opportunities” (Iran-Nejad, 2013, p. 53). Given the appropriate environment, learners are able to engage in the processes associated with Bransford and colleague’s principles that enable them to learn with rich understanding.

### Characterizing Biofunctional Classrooms

Biofunctional approaches to education are essentially constructivist in nature, not entirely unlike Piagetian methods. Learners must reflect on his or her intuitive thought processes in order for learning to occur. Essentially, the knowledge one learns represents a reorganization process, relying on existing knowledge and dynamic self-regulation, from within the learner’s own brain and biological subsystems. This is total reliance on one’s internal resources rather than outside resources, i.e. external stimuli. This is in line with Piagetian reorganization processes of assimilation and accommodation. However, this process of constructing knowledge through reorganization should be distinguished from other social-constructivist, or Vygostkian methods. Social-constructivist methods rely on the body’s responses to external stimuli in the construction of new knowledge. Both produce knowledge, but biofunctional classrooms promote learning that results from processes entirely within the learner and can be reduced to interactions among the learner’s biological systems. Social-constructivist classrooms promote learning that results from internal and external processes and can be reduced to interactions between the person and their environment.

### Promoting Awareness of Insights in a Natural Environment

The nature of a classroom is, in many cases, very unnatural. Classrooms are often not aligned with the natural environments associated with the subject matter being presented. However, a key theme of creating biofunctional classrooms is helping students to recognize and keep track of their intuitions and insights in a natural, biologically facilitated manner (Iran-Nejad and Gregg, 2001). A way of doing this could be to record one’s insights in a journal, as suggested by Stewart et al. (2008), in the context of a history classroom. Specifically, they suggest recording insights that occur through the exploration of counterfactual historical topics, which promote meaningful, personal connections to the subject matter. This practice runs counter to the teacher acting as a “giver” of information. A key feature of this type of biofunctional approach is that the learner’s biological subsystems are engaged during the process of understanding, rather than relying on symbolic interactions with the outside world (Iran-Nejad, 2000).

Another example could involve students designing a demonstration on a particular state of matter, and recording the moments when they come to a certain realization, sometimes called revelations or “clicks of understanding” (Iran-Nejad, 2000). These moments often produce sparks of enthusiasm and excitement. It is the teacher’s duty to help students identify these moments so that they are able to reflect on what they have learned. Through reflection, students are able to process what they have learned. Within a biofunctional classroom, this cycle of understanding occurs entirely within the student, rather than from the teacher trying to force learning on the students. This mode of learning is not entirely unlike other constructivist methods of learning, but a distinguishing characteristic of biofunctional learning environments is the recognition of the dynamic biological mechanisms at work within the learner. In this sense, biofunctional learning environments are aligned with constructivist methods of learning, but not entirely aligned with social-constructivist methods.

Another method, suggested by Gregg and Sekeres (2008) focuses on using wholetheme approaches to class discussions. Wholetheme approaches to education make use of the holistic aspects of the brain by focusing on whole themes of knowledge (the big picture), rather than forcing students to analyze and internalize bits and pieces of concepts (Iran-Nejad, 1994; Saleh and Iran-Nejad, 1995). This enables the learner to tap into his or her intuitive and biofunctional resources rather than trying to remember disconnected facts. The teacher, in this case, focuses on using probing questions to allow students to naturally engage in a state of reflection on their personal intuitions. In this example, the teacher facilitates the discussion, but does not dominate the discussion. Instead, the students’ thoughts and insights are the focus. Other approaches may also provoke revelation-learning opportunities within the learner, but wholetheme methods capitalize on learners’ ongoing biofunctional activity in order to help the learner dynamically self-regulate and come to deep understanding of core knowledge themes.

## Conclusion

This paper argues that traditional educational methods rely too much on superficial metaphors, rather than focusing on the embodied learning resources inherent to the learner. For learning environments to be effective, from a biofunctional perspective, they must be constructed in such a way that they align with the principles of learning, but also allow the principles to be grounded in the learner’s biological functioning. Several examples have been put forth to illustrate 1st person educational approaches that enable students to engage in biofunctional understanding. These examples and other, similar approaches must involve the principles of learning, i.e. “engaging prior understanding”, “organizing prior experiences”, and “self-monitoring”, working together as a dynamic system in a wholetheme global coherence context, rather than in isolated building blocks of piecemeal cognition. These principles of learning, in essence, must be biologically grounded for real learning to occur. Furthermore, this dynamic biological system is the intrinsic facilitator of students’ learning, in which learners function from a natural state, rather than the teacher facilitating students’ learning through artificial means.

## Conflict of Interest Statement

The author declares that the research was conducted in the absence of any commercial or financial relationships that could be construed as a potential conflict of interest.
